# Toxicity of the Pesticides Imidacloprid, Difenoconazole and Glyphosate Alone and in Binary and Ternary Mixtures to Winter Honey Bees: Effects on Survival and Antioxidative Defenses

**DOI:** 10.3390/toxics10030104

**Published:** 2022-02-23

**Authors:** Elisa Pal, Hanine Almasri, Laurianne Paris, Marie Diogon, Maryline Pioz, Marianne Cousin, Déborah Sené, Sylvie Tchamitchian, Daiana Antonia Tavares, Frédéric Delbac, Nicolas Blot, Jean-Luc Brunet, Luc P. Belzunces

**Affiliations:** 1 INRAE, UR 406 A&E, Laboratoire de Toxicologie Environnementale, F-84000 Avignon, France; elisa.pal@fabi.up.ac.za (E.P.); hanine.almasri@gmail.com (H.A.); maryline.pioz@inrae.fr (M.P.); marianne.cousin@inrae.fr (M.C.); deborah.sene@laposte.net (D.S.); sylvie.tchamitchian@neuf.fr (S.T.); daianatavareszoo@gmail.com (D.A.T.); jean-luc.brunet@inra.fr (J.-L.B.); 2 CNRS, Laboratoire Microorganismes, Génome et Environnement, Université Clermont Auvergne, F-63000 Clermont-Ferrand, France; laurianne.paris@uca.fr (L.P.); marie.diogon@uca.fr (M.D.); frederic.delbac@uca.fr (F.D.); nicolas.blot@uca.fr (N.B.)

**Keywords:** honey bees, pesticide mixtures, physiological alterations, oxidative stress

## Abstract

To explain losses of bees that could occur after the winter season, we studied the effects of the insecticide imidacloprid, the herbicide glyphosate and the fungicide difenoconazole, alone and in binary and ternary mixtures, on winter honey bees orally exposed to food containing these pesticides at concentrations of 0, 0.01, 0.1, 1 and 10 µg/L. Attention was focused on bee survival, food consumption and oxidative stress. The effects on oxidative stress were assessed by determining the activity of enzymes involved in antioxidant defenses (superoxide dismutase, catalase, glutathione-*S*-transferase, glutathione reductase, glutathione peroxidase and glucose-6-phosphate dehydrogenase) in the head, abdomen and midgut; oxidative damage reflected by both lipid peroxidation and protein carbonylation was also evaluated. In general, no significant effect on food consumption was observed. Pesticide mixtures were more toxic than individual substances, and the highest mortalities were induced at intermediate doses of 0.1 and 1 µg/L. The toxicity was not always linked to the exposure level and the number of substances in the mixtures. Mixtures did not systematically induce synergistic effects, as antagonism, subadditivity and additivity were also observed. The tested pesticides, alone and in mixtures, triggered important, systemic oxidative stress that could largely explain pesticide toxicity to honey bees.

## 1. Introduction

The honey bee *Apis mellifera* is a pollinator insect of agro-environmental and economic importance [1,2]. It improves the production of approximately 75% of global crops [3]. In 2009, the worldwide economic value of insect pollination for agriculture was estimated at EUR 153 billion per year, which represented 9.5% of the value of the world agricultural production used for human food in 2005 [4]. However, despite the development of beekeeping, a constant decline in honey bee populations has been observed in numerous countries since the beginning of the 20th century [1,5,6,7]. This phenomenon is multicausal, and several factors that contribute to this decline have been identified [8,9]. During foraging, in a radius up to 12 km around the hive [10], honey bees are in contact with a large variety of environmental stressors, including pesticides and pathogens, and there seems to be a consensus that pesticides and pathogens represent the main contributors to colony decline [6].

A large number of pesticide residues can be found in apicultural matrices such as honey, pollen and beeswax [11,12,13,14,15,16]. Pesticides have been shown to have strong impacts on all ecosystems, and many pesticides, particularly insecticides, have been shown to affect bees [17,18,19]. Pesticides may elicit both lethal and adverse sublethal effects after acute or chronic exposure of bees either directly, during or after a plant protection treatment, or indirectly by the consumption of food (nectar and pollen) contaminated with pesticide residues [17,20,21].

Pesticides can act not only alone but also in combination to induce synergistic effects [22,23,24,25,26,27]. However, research on the synergistic action of pesticide mixture toxicity in honey bees is relatively scarce. The majority of studies are focused on possible synergistic effects on mortality in summer bees between pyrethroid insecticides and azole fungicides [22,23,24,28,29], neonicotinoid and pyrethroid insecticides [25,30], and between neonicotinoid insecticides and azole fungicides [31,32,33,34]. However, substances belonging to the three main classes of pesticides, i.e., herbicides, fungicides and insecticides, have been detected in beehives [35,36,37]. Thus, it is crucial to investigate the combined effects of mixtures of pesticides from different classes at environmentally relevant concentrations.

In a previous study [38], we showed that binary and ternary mixtures of the insecticide imidacloprid, the fungicide difenoconazole and the herbicide glyphosate induced a high toxicity in winter honey bees at environmental concentrations. It was surprising to see that these substances induced toxicity by a systemic action, which strongly suggests that nonspecific effects might be involved in the induction of toxicity. In addition, the modulation of glutathione-S-transferase (GST) and glucose-6-phosphate dehydrogenase (G6PDH) suggested the involvement of oxidative stress in the toxicity of the pesticide mixtures. Thus, to better understand the action of pesticide mixtures, we conducted a mechanistic study that aimed to (i) confirm the results previously obtained on binary and ternary mixtures of imidacloprid, difenoconazole and glyphosate and (ii) investigate the toxicological pathways involved in the mode of action of the mixtures. As a first approach, we focused our attention on oxidative stress, a general phenomenon involved in health degradation [39]. We also considered a fourth exposure level (0.01 µg/L) to better investigate the domain of low doses. Imidacloprid is a neonicotinoid insecticide that acts as an agonist of nicotinic acetylcholine receptors and induces hyperactivation of cholinergic neurons [40]. Difenoconazole is a triazole fungicide belonging to the family of azole active substances that act as ergosterol biosynthesis inhibitors (EBI fungicides). These fungicides alter the structure of the fungal cell membrane by inhibiting ergosterol synthesis [41]. Glyphosate is a phosphoglycine herbicide that blocks the synthesis of plant aromatic amino acids by inhibiting *5*-enolpyruvylshikimate-*3*-phosphate synthase (EPSPS), an enzyme involved in the shikimate pathway [42]. We selected active substances from the three main classes of pesticides to match agricultural practices, for which herbicides, fungicides and insecticides represent 52.5%, 23% and 17% of the total mass of pesticide sold during the 1990–2019 period, respectively [43]. Glyphosate difenoconazole and imidacloprid were chosen as representatives of these three classes of pesticides because they are among the pesticides frequently detected in beehive matrices. Imidacloprid, along with its metabolite 6-chloronicotinic acid, is the most abundant pesticide in beehive matrices in French apiaries; glyphosate is the most dominant herbicide worldwide, and difenoconazole is authorized for use during full bloom [38]. This study was focused on winter bees because they maintain the colony during winter and are therefore important for the start of colony development during the spring. Moreover, their particular longevity makes them exposed during a long period of time. Here, we considered the effects of pesticides on bee longevity, food intake and physiology by exploring both oxidative damage and changes in antioxidant defenses.

## 2. Materials and Methods

### 2.1. Materials

Antipain, aprotinin, leupeptin, pepstatin A, soybean trypsin inhibitor, monobasic and dibasic sodium and potassium phosphates (NaH_2_PO_4_, KH_2_PO_4_, K_2_HPO_4_ and Na_2_HPO_4_), sodium chloride (NaCl), potassium chloride (KCl), Triton X-100, reduced L-glutathione (GSH), glucose-*6*-phosphate (G6P), NADPH, NADP^+^, ethylenediaminetetraacetic acid (EDTA), xanthine, xanthine oxidase, nitroblue tetrazolium (NBT), hydrogen peroxide (H_2_O_2_), tert-butyl hydroperoxide (TBHP), glutathione reductase, 1-chloro-2,4-dinitrobenzene (CDNB), trishydroxymethyl-aminomethane base (Tris), sodium bicarbonate (NaHCO_3_), hydrochloric acid (HCl), iron(III) chloride (FeCl_3_), *4*-(*2*-hydroxyethyl)-1-piperazineethanesulfonic acid (HEPES), *2*,*4*-dinitrophenylhydrazine (DNPH), nonidet P-40 (NP-40), magnesium chloride (MgCl_2_), sodium dodecyl sulfate (SDS) and bovine serum albumin (BSA) were obtained from Sigma Aldrich^®^ (Saint Quentin Fallavier, France). Imidacloprid (CAS No 138261-41-3), difenoconazole (CAS No 119446-68-3) and glyphosate (CAS No. 1071-83-6) (98% purity) were purchased from Cluzeau Info-Labo (Sainte-Foy-la-Grande, France). Anti-DNP antibody (clone 9H8.1) was obtained from Millipore™ (Guyancourt, France), and goat anti-mouse IgG HRP conjugate was obtained from Promega (Charbonnières, France). The Clarity^TM^ Western ECL substrate was purchased from Bio–Rad (Roanne, France).

### 2.2. Honey Bees

In January, *A. mellifera* honey bees were collected from three colonies, monitored for their health status, located at the experimental apiary of the *Abeilles & Environnement* (Bees & Environment) Research Unit of Avignon INRAE Research Centre (southern France). The age of the bees was not controlled to reflect the winter bee population. Bees were slightly anesthetized with a light carbon dioxide flow, mixed and randomly distributed into laboratory cages (Pain type, 6 × 8.5 × 10 cm) in groups of 30 individuals per cage. The cages were then placed in the dark in an incubator at controlled conditions (30 °C ± 2 °C; 60% ± 10% relative humidity) until the end of the experiment. During the first 24 h of the experiment, the bees were provided with water and candy (Apifonda^®^) ad libitum, and the dead honey bees were removed and replaced. To optimize hygiene conditions, a sheet of filter paper was placed on the bottom of the cages and replaced daily.

### 2.3. Exposure to Pesticides

Honey bees were exposed for 24 h per day for 16 days by feeding a 60% (*w*/*v*) sucrose syrup containing 1% (*v*/*v*) Provita’ Bee^®^ (ATZ Dietetics, Mas-Cabardès, France) protein preparation and the insecticide imidacloprid, the fungicide difenoconazole and the herbicide glyphosate alone or in mixtures. For each pesticide, five concentrations were tested: 0 (control), 0.01, 0.1, 1 and 10 μg/L (equivalent to 0, 0.0083 0.083, 0.813 and 8.130 µg/kg, respectively, calculated with a sucrose solution density of 1.23 ± 0.02 g/L (*n* = 10)). Concentrations were consistent with the residual contamination found in honey, pollen and wax [11,15,44,45,46,47,48]. Six experimental groups of exposure were investigated: the control group (C); the insecticide alone (I); the fungicide alone (F); the herbicide alone (H); the binary mixtures of insecticide + fungicide (IF), insecticide + herbicide (IH) and herbicide + fungicide (HF); and the ternary mixture of insecticide + herbicide + fungicide (IHF). For each group and at each exposure concentration, 14 replicates of 30 honey bees were exposed. Syrup consumption and mortality were recorded daily until the end of the experiment. For pesticide mixtures, all pesticides were used at the same concentration. The pesticide solutions were prepared in water and DMSO and stored at −20 °C until use. The sucrose feed solutions were prepared daily and contained 60% (*w*/*v*) sucrose, pesticides (or no pesticides in the control) and 0.1% (*v*/*v*) DMSO. The pesticide concentrations were confirmed by GC–MS/MS following two analytical methods [49,50]. For each concentration, the relative standard deviations (RSD) compared to the nominal concentrations were less than 8%.

### 2.4. Analysis of Physiological Life History Traits

Variations in physiological life history traits were analyzed after 16 days of exposure to pesticides in living bees. The period of 16 days was chosen because some treatments drastically compromised bee survival, and it was necessary to have a sufficient number of living bees to conduct physiological analyses. The physiological traits were assessed in tissues in which they are relevant and where their biological activity is particularly high as previously described [51]. Physiological traits were analyzed in surviving bees exposed to pesticides at concentrations of 0.1 and 1 μg/L. To avoid animal suffering, tissue samples were collected after anesthesia and decapitation of bees. For each bee, the head, midgut and abdomen (without the intestine) were sampled, immediately quick-frozen in liquid nitrogen, weighed and stored at −80 °C until analysis. The tissues were ground in extraction medium with Qiagen^®^ TissueLyser II (30 Hz; 3 periods of 30 sec at 30 sec intervals) to make a 10% (*w*/*v*) extract. The extraction medium consisted of 10 mM NaCl, 1% (*w*/*v*) Triton X-100 and 40 mM sodium phosphate at pH 7.4 and contained protease inhibitors (2 µg/mL antipain, leupeptin and pepstatin A; 25 units/mL aprotinin; and 0.1 mg/mL soybean trypsin inhibitor) [52]. Tissue extracts were centrifuged at 4 °C for 20 min at 15,000× *g*, and the supernatants were collected for analysis [53]. For each treatment, seven repetitions were performed and assayed in triplicate, and each sample corresponded to tissues pooled from three bees per cage sampled in seven cages.

Physiological traits were spectrophotometrically assayed at 25 °C on different organs of the same bees. For traits, a blank assay was performed to assess the background spontaneous activity. Activities of physiological traits were expressed as variation of absorbance units per minute and were standardized by their respective control activities. The activities of glutathione-*S*-transferase (GST), catalase (CAT) and superoxide dismutase (SOD) were measured in both the head and midgut. The activities of glutathione reductase (GR) and glutathione peroxidase (GPox) were measured in the head, and the activity of glucose-*6*-phosphate dehydrogenase (G6PDH) was measured in the abdomen and midgut. Abdomen and midgut G6PDH activities were determined by continuously following the formation of NADPH at 340 nm. The reaction medium contained 10 mM MgCl_2_, 1 mM glucose-*6*-phosphate, 0.5 mM NADP^+^ and 100 mM Tris-HCl pH 7.4. Head and midgut GST activities were determined by measuring the conjugation of GSH to CDNB at 340 nm. The reaction medium contained 1 mM EDTA, 2.5 mM GSH, 1 mM CDNB and 100 mM Na/K phosphate at pH 7.4 [54]. SOD activity was determined at 560 nm in a reaction medium containing 0.1 mM EDTA, 0.1 mM xanthine, 0.025 mM nitroblue tetrazolium (NBT), 8.33 mU/mL xanthine oxidase and 50 mM sodium phosphate/carbonate at pH 7.8. Head GPox activity was assayed using *tert*-butyl hydroperoxide (TBHP) as the substrate. The generated oxidized glutathione (GSSG) was reduced in the presence of NADPH by GR to generate GSH and NADP^+^. The conversion of NADPH into NADP^+^ was followed at 340 nm. The reaction medium contained 1 mM EDTA, 0.2 mM TBHP, 0.85 mM GSSG, 0.16 mM NADPH, 0.25 U/mL GR and 50 mM Na/K phosphate at pH 7.4. Head GR activity was determined at 340 nm by the conversion of NADPH to NADP^+^. The reaction medium contained 1 mM EDTA, 0.85 mM GSSG, 0.16 mM NADPH and 50 mM Na/K phosphate at pH 7.4 [55]. The decomposition of H_2_O_2_ by CAT was measured at 240 nm. The reaction medium contained 10 mM H_2_O_2_ and 100 mM sodium phosphate at pH 7.0 [56]. All enzymatic reactions were followed on a TECAN F500 spectrophotometer.

### 2.5. Lipid Peroxidation

Lipid peroxidation was assessed by the measurement of thiobarbituric acid-reactive substances (TBARS). The malondialdehyde (MDA) content determined with thiobarbituric acid was considered representative of overall lipid peroxidation [57]. MDA was fluorometrically assayed with the TCA method (TBARS (TCA method) assay kit no 700870, Cayman Chemical, MI, USA) at λ_exc_ = 530 nm and λ_em_ = 550 nm. For each modality, nine extracts of three midguts per extract (*n* = 9) were assayed in triplicate.

### 2.6. Quantification of Protein Carbonylation

According to Paris et al. (2017), proteins were extracted on ice by crushing the midguts in radioimmunoprecipitation assay (RIPA) buffer (150 mM NaCl, 0.5% sodium deoxycholate, 1% NP-40, 0.1% SDS, and 50 mM Tris-HCl at pH 8.0) supplemented with 1 mM PMSF to obtain a 10% (*w*/*v*) extract by means of a Eurostar digital IKA stirrer (Labortechnik). For each modality, 12 midguts (*n* = 12) were assayed in triplicate. The extracts were incubated on ice for 15 min, vortexed every 5 min, and then centrifuged at 4 °C for 15 min at 14,000× *g*. The supernatant was kept for analysis. The protein content was assayed with Bradford’s method [58] using the Coomassie Plus™ (Bradford) assay kit (Thermo Scientific, Rockford, USA). The measurements were performed in triplicate after a 1/40 dilution. To assess the carbonylation rate of proteins, carbonylated BSA was used as a standard according to Yoo and Regnier (2004): first, 10 mg of BSA was solubilized in 900 µL of solubilization buffer (250 mM ascorbic acid and 1 mM FeCl_3_). Then, 100 µL of oxidation solution (100 mM KCl, 10 mM MgCl_2_ and 50 mM HEPES at pH 7.4) was added. The reaction was stopped by adding EDTA [59]. The quantification of protein carbonylation was performed according to Paris et al. (2017). Briefly, 18 µg of global proteins or the BSA standard was denatured in SDS, derivatized with DNPH and neutralized in Tris-base. The carbonylated proteins were slot-blotted on a polyvinylidene difluoride (PVDF) membrane with a slot blotter (PR 600 slot-blot, Hoefer). The membranes were incubated overnight at 4 °C with diluted (1/2000) anti-DNP antibody (clone 9H8.1, Millipore^TM^) and then for 1 h at 25 °C with the diluted (1/2500) secondary antibody coupled to horseradish peroxidase (goat anti-mouse IgG HRP conjugate, Promega). Detection was performed by chemiluminescence (Clarity™ Western ECL Substrate, Bio–Rad), and the signal was analyzed with a ChemiDOC™ MP system analyzer (Bio–Rad).

### 2.7. Statistical Analysis

Statistics were performed using RStudio version 1.1.463 statistical software. Survival analyses were performed using the packages *survival* and *survminer*, and the Kaplan–Meier method was used followed by a post hoc test for comparison of survival between treatments. The Kruskal–Wallis test, followed by pairwise comparisons using the Wilcoxon rank test (with Benjamini–Hochberg correction), was used to compare the cumulative individual food consumption between treatments. The effects of treatments on enzymatic activities, lipid peroxidation and protein carbonylation were determined by ANOVA followed by Tukey’s HSD test or by a Kruskal–Wallis test followed by post hoc Dunn’s test (with Benjamini–Hochberg correction using the *agricolae* package). Principal component analyses (PCAs) were performed using the *FactoMineR* package to distinguish the different treatments according to their effects on physiological markers.

### 2.8. Mode of Interaction between Pesticides

The mode of interaction between pesticides (additive, antagonistic and synergistic) was evaluated by the interaction ratio (IR) (Table S1) described by Pigott et al. [60] and used to study interactions between active substances in pesticide mixtures in the honey bee [38]:

$$\mathrm{IR}=\frac{\left(Mix-C\right)}{\sum_{n=0}^{2-3}(P_{n}-C)}$$

where *Mix* represents the crude mortality of the mixture, *C* is the mortality of the control, (*Mix* − *C*) is the mortality of the pesticide mixture (binary or ternary) corrected by the control mortality, and $\overset{2-3}{\underset{n=0}{\sum }}(P_{n}-C)$ represents the sum of the mortalities induced by each pesticide (*n*) in the mixture corrected by the control mortality, which corresponds to the theoretical expected mortality of the mixture. A value of IR = 1 reflects a pure additive effect. However, considering variations in the effects, an IR is considered = 1 when 0.80 ≤ IR ≤ 1.20. When IR > 1.20, the interaction is synergistic. For IR < 1, four cases were distinguished (Table S1): (i) When the mortality of the mixture was lower than the mortality of the lowest toxic substance alone, the interaction could be considered purely antagonistic. (ii) When the toxicity of the mixture was higher than the mortality of the most toxic substance but below the expected mortality, the interaction was considered subadditive. In this case, it was not possible to speak in terms of antagonism because the effect of the mixture was higher than the effect of each substance alone. (iii) When the effect of the mixture ranged between the effect of the least toxic substance and the effect of the most toxic substance, the interaction was also considered subadditive. In this case, it was also not possible to speak in terms of antagonism because compared to the most toxic substance, antagonism could be considered, but compared to the lowest toxic substance, synergy could also be considered. (iv) When the mixture induced a mortality similar to that of each pesticide, the effect of the mixture was considered independent (Table S1). However, this case was not observed.

## 3. Results

### 3.1. Chronic Toxicity of Pesticides Alone or in Combination

**Figure 1:**
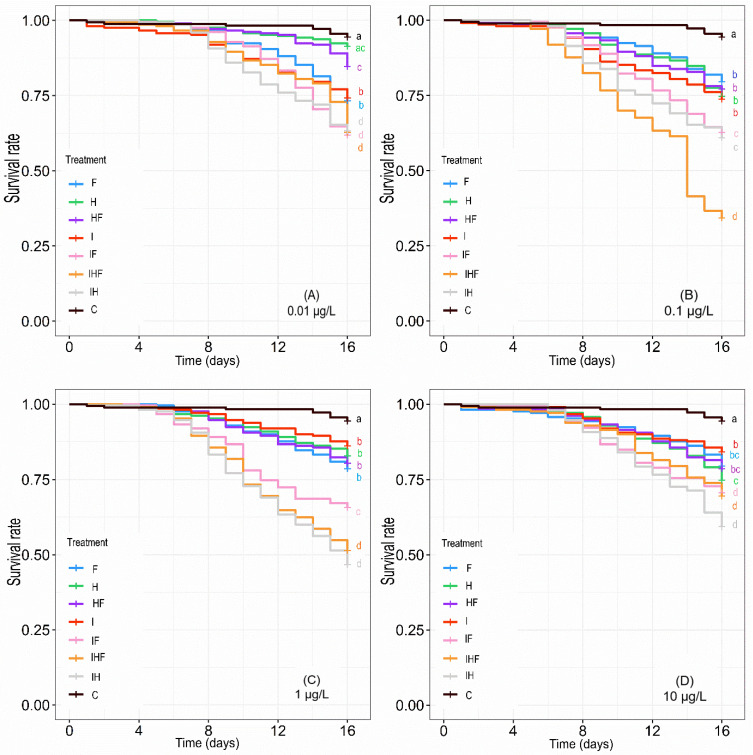
Effects of pesticides alone or in mixtures on honey bee survival. Winter honey bees were orally exposed to food containing no pesticides (C), imidacloprid (I), difenoconazole (F), glyphosate (H), imidacloprid + difenoconazole (IF), imidacloprid + glyphosate (IH), glyphosate + difenoconazole (HF), or imidacloprid + glyphosate + difenoconazole (IHF) at concentrations of 0.01 µg/L (**A**), 0.1 µg/L (**B**), 1 µg/L (**C**) and 10 µg/L (**D**). The survival rate was followed until day 16 of exposure, at which bees were sampled for physiological analyses. The data represent the mean proportion of surviving honey bees. The mortalities from 14 replicates of 30 bees per treatment were analyzed using the Kaplan–Meier method followed by a post hoc test for comparison of survival between treatments. Data with different letters are significantly different (*p* < 0.05).

Bees were exposed for 16 days to three pesticides at four different concentrations (0.01 µg/L, 0.1 µg/L, 1 µg/L and 10 µg/L), alone or in mixtures, and their survival rate was recorded every day (Figure 1). In general, at all concentrations, the survival rate of the honey bees exposed to pesticides was significantly lower than that of the control, and the highest toxicities were observed at the intermediate concentrations of 0.1 and 1 µg/L. In addition, except for HF, the toxicity of the mixtures was higher than that of the individual pesticides. For each exposure condition, the highest toxicity, expressed as corrected mortality, was observed with IF (29.8%), IH (27.4%) and IHF (29.1%) at 0.01 µg/L; IHF (57.6%) at 0.1 µ/L; IH (46.2%) and IHF (40.5%) at 1 µg/L; and IF (20.9%), IH (32.1%) and IHF (21.9%) at 10 µg/L (Table S2).

The mode of interaction between pesticides was evaluated by the IR, which corresponds to the ratio between the effects induced by the mixture to the expected effects of the mixture, which is the sum of the effects induced by each component of the mixture alone (Figure 1 and Table S1). Antagonistic interactions between pesticides were observed with the binary mixtures containing the herbicide and fungicide (HF), with marked antagonism observed for HF0.01 and HF1 and slight antagonism observed for HF0.1 and HF10. Subadditive interactions were observed with the binary mixtures IF0.01 and IH0.1 and with the ternary mixture IHF0.01 and IHF10. Additive interactions were observed with the binary mixtures IF0.01, IH0.01, IF0.1, IH0.1, IHF1 and IF10 and with the ternary mixture IHF1. Interestingly, synergistic interactions were observed for 4 out of the 16 mixtures, with the binary mixtures IF1, IH1, and IH10 and with the ternary mixture IHF 0.1.

### 3.2. Effects of Pesticides on Feeding Behavior

**Figure 2:**
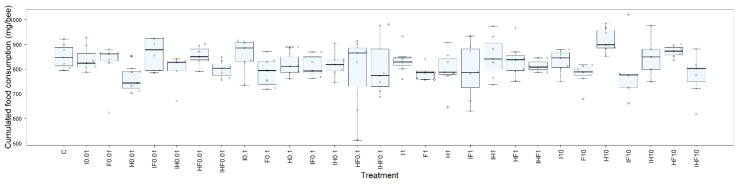
Effect of exposure to pesticides on food consumption. Winter honey bees were orally exposed to food containing no pesticides (C), imidacloprid (I), difenoconazole (F), glyphosate (H), imidacloprid + difenoconazole (IF), imidacloprid + glyphosate (IH), glyphosate + difenoconazole (HF), or imidacloprid + glyphosate + difenoconazole (IHF) at concentrations of 0.01, 0.1, 1 and 10 µg/L. Food consumption was evaluated daily during the 16-day period. Box plots represent the cumulative individual consumption (mg/bee) at day 16 as determined from 14 cages of 30 bees per treatment. Statistical analyses were performed using the Kruskal–Wallis test followed by pairwise comparisons using the Wilcoxon rank sum test with Benjamini–Hochberg correction. The numbers after the abbreviations of each treatment refer to the concentrations of the pesticides in the food. Asterisks indicate significant differences from the control group (* *p* ≤ 0.05).

The influence of pesticide treatments on the feeding behavior of honey bees was followed by measuring the daily food consumption (Figure 2). The individual cumulative food consumption was used to detect possible differences between treatments. As a general feature, honey bees exposed to pesticides consumed an equal amount of food compared to that consumed by unexposed bees, except for the bees of H0.01 that consumed relatively less food than controls (753.7 mg/bee and 852.3 mg/bee, respectively (Table S3)). When comparing the cumulative individual food consumption between different doses of the same treatment, honey bees exposed to glyphosate consumed significantly less food when exposed at 0.01 µg/L than when exposed at 10 µg/L (753.7 mg/bee and 917.3 mg/bee, respectively (Table S3)). Because there are no significant differences in food consumption between bees exposed to pesticides alone and bees exposed to pesticide mixtures (except for H0.01), the quantity of pesticide ingested cannot explain the effects observed at the same level of exposure. This is particularly true for mixtures exhibiting a synergistic interaction (IHF0.1, IF1, IH1 and IH10) for which the consumption of the bees exposed to pesticide mixtures is either less than or close to those of bees exposed to pesticides alone (Tables S1 and S3).

### 3.3. Variations in Physiological Life History Traits by Pesticides

**Figure 3:**
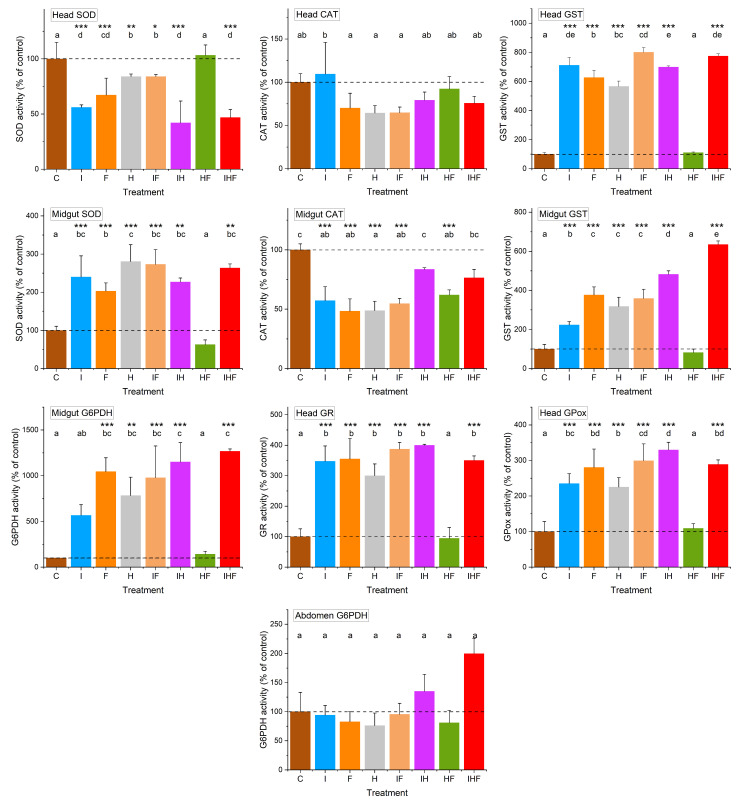
Effects of exposure to pesticides at 1 µg/L on antioxidant defenses. Winter honey bees were orally exposed to food containing no pesticides (C), imidacloprid (I), difenoconazole (F), glyphosate (H), imidacloprid + difenoconazole (IF), imidacloprid + glyphosate (IH), glyphosate + difenoconazole (HF), or imidacloprid + glyphosate + difenoconazole (IHF) at a concentration of 1 µg/L. On day 16, enzymes involved in antioxidant defenses were assayed in the head, midgut and abdomen of bees. SOD, superoxide dismutase; CAT, catalase; GST, glutathione-*S*-transferase; G6PDH, glucose-*6*-phosphate dehydrogenase; GR, glutathione reductase; GPox, glutathione peroxidase. The data represent the means of tissue activities from 14 repetitions performed in triplicate and are expressed as percentages of the mean control value. Data with different letters are significantly different (*p* < 0.05). Asterisks indicate significant differences from the control group: * *p* < 0.05; ** *p* < 0.01; *** *p* < 0.001. The dotted lines indicate the control levels.

**Figure 4:**
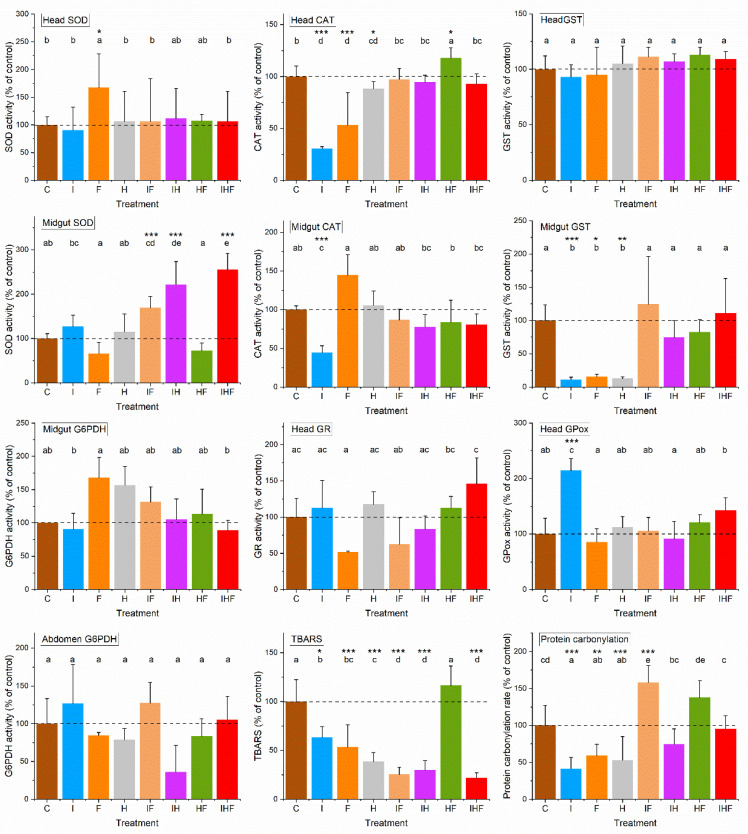
Effects of exposure to pesticides at 0.1 µg/L on antioxidant defenses and oxidative damage. Winter honey bees were orally exposed to food containing no pesticides (C), imidacloprid (I), difenoconazole (F), glyphosate (H), imidacloprid + difenoconazole (IF), imidacloprid + glyphosate (IH), glyphosate + difenoconazole (HF), or imidacloprid + glyphosate + difenoconazole (IHF) at a concentration of 0.1 µg/L. On day 16, enzymes involved in antioxidant defenses were assayed in the head, midgut and abdomen of bees, and lipid peroxidation (TBARS) and protein carbonylation were assessed. SOD, superoxide dismutase; CAT, catalase; GST, glutathione-*S*-transferase; G6PDH, glucose-*6*-phosphate dehydrogenase; GR, glutathione reductase; GPox, glutathione peroxidase; TBARS, thiobarbituric acid-reactive substances. The data represent the means of tissue activities from 14 repetitions performed in triplicate and are expressed as percentages of the mean control value. Data with different letters are significantly different (*p* < 0.05). Asterisks indicate significant differences from the control group: * *p* < 0.05; ** *p* < 0.01; *** *p* < 0.001. The dotted lines indicate the levels of controls.

The variations in physiological life history traits were analyzed after 16 days of exposure to pesticides alone or in mixtures (Figure 3 and Figure 4). Changes at 0.1 and 1 µg/L were preferred for analysis because these groups exhibited the highest mortality rates, and the pesticide concentrations were environmentally relevant. Analyses were focused on oxidative stress by analyzing antioxidative defenses.

Generally, at 1 µg/L, there was a large change in enzyme activities involved in antioxidant defenses (Figure 3) (Table S5). No change was observed in the activity of CAT in the head and G6PDH in the abdomen of honey bees. Decreased activity was observed for CAT in the gut in five out of seven exposure groups (excluding IH and IHF) and for SOD in the head of bees of all exposure groups except HF. For head and gut GST, GR and GPox activities and gut SOD activity, an increase was observed in all exposure groups except HF. Decreased activity was also observed for gut G6PDH in almost all exposure groups except I and HF.

The bees exposed to pesticides at a concentration of 0.1 µg/L exhibited relatively complex changes in physiological life history traits (Figure 4) (Table S4). Four categories of variations in enzyme activity were observed: (i) no change at all (head GST and GR activities and abdomen and gut G6PDH activities); (ii) increased activities (head SOD activity with F, head GPox activity with I and gut SOD activity with IF, IH and IHF); (iii) decreased activities (gut GST activity with I, F and H and gut CAT activity with I); and (iv) increased and decreased activity depending on exposure conditions (the CAT activity in the head decreased with I, F and H and increased with HF). It appeared that the exposure cases for which the lowest number of antioxidant enzymes were affected corresponded to binary and ternary pesticide mixtures. However, in contrast with the concentration of 1 µg/L, for which a large change in antioxidant enzyme activities was observed, indications of oxidative stress were less obvious at the concentration of 0.1 µg/L, especially for the binary and ternary mixtures. Thus, we investigated the damage caused by oxidative stress at 0.1 µg/L by analyzing lipid peroxidation, reflected by TBARS, and protein oxidation, reflected by amino acid carbonylation (Figure 4). For TBARS, a decrease was observed with all exposure conditions, except with HF. For protein oxidation, a decrease was observed, except with IH, HF and IHF, which induced values similar to that of the control, and with IF, for which the carbonylation rate represented 158% of that of the control (28.2 ± 4.1% carbonylated proteins/mg of tissue for IF and 17.9 ± 4.8% carbonylated proteins/mg of tissue for the control (Table S6)).

**Figure 5:**
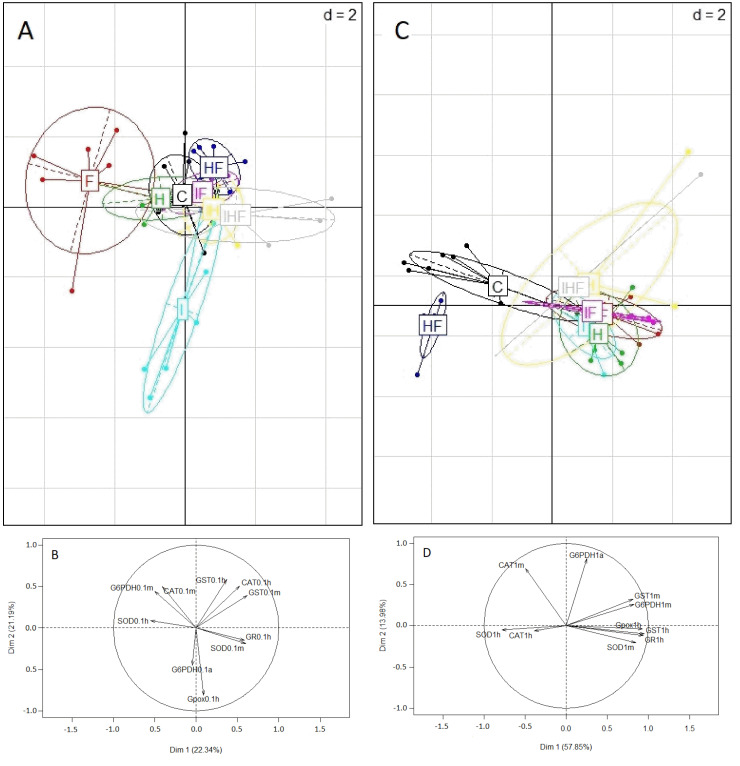
Effects of exposure to pesticides on the physiological state of winter honey bees. Winter honey bees were orally exposed to food containing no pesticides (C), imidacloprid (I), difenoconazole (F), glyphosate (H), imidacloprid + difenoconazole (IF), imidacloprid + glyphosate (IH), glyphosate + difenoconazole (HF), or imidacloprid + glyphosate + difenoconazole (IHF) at concentrations of 0.01, 0.1, 1 and 10 µg/L. On day 16, enzymes involved in antioxidant defenses were assayed. SOD, CAT and GST were measured in the head (h) and midgut (m). GPox and GR were measured in the head (h), and G6PDH was measured in the midgut (m) and abdomen (a). A multiple marker approach was performed to analyze the effects of these pesticides at 0.1 and 1 µg/L on oxidative stress. Principal component analyses (PCAs) (A and C) provide visual representations of the physiological states of honey bees exposed to the three pesticides individually or in binary and ternary mixtures at 0.1 µg/L (**A**) and 1 µg/L (**C**). The correlation circles (**B**,**D**) indicate the significance of the enzymes in the PCA representations in honey bees exposed to the pesticides individually or in binary and ternary mixtures at 0.1 µg/L (**B**) and 1 µg/L (**D**). SOD, superoxide dismutase; CAT, catalase; GST, glutathione-*S*-transferase; G6PDH, glucose-*6*-phosphate dehydrogenase; GR, glutathione reductase; GPox, glutathione peroxidase.

PCA was conducted to distinguish the different treatments according to their effects on the 10 studied physiological markers (Figure 5A,C). The correlation circles (Figure 5B,D) indicate which enzymes had the largest influence on the determination of the physiological state of honey bees following exposure to each treatment. At 0.1 µg/L, the two axes accounted for 44.2% of the total dataset variation (Figure 5A,B). Therefore, this PCA did not enable the distinction of antioxidant enzyme activities. This complex representation was in accordance with our hypothesis of a relatively complex pattern of change in the physiological life history traits at an exposure level of 0.1 µg/L. At 1 µg/L (Figure 5C,D), the two axes of the PCA accounted for 71.82% of the total dataset variation; therefore, this PCA sufficiently distinguished the activities of the enzymes. The enzymes were clearly separated into two groups; the first group was on the right of the correlation circle (Figure 5D) and corresponded to the markers whose activities increased after exposure to pesticides (head GST, GR, and GPox activities and midgut SOD, GST and G6PDH). The second group was on the left of the correlation circle and corresponded to the enzymes exhibiting a decrease in activity after exposure (head SOD and midgut CAT). In the midgut, GST and G6PDH activities were positively correlated with each other. However, these markers were independent of CAT activity in the same organ. In the head, GPox, GST and GR activities were positively correlated, while G6PDH activity was independent of that of CAT. The presence of these two clearly separated groups appeared to have the largest influence on distinguishing the control and HF treatments from the other treatments in the PCA plot at the exposure level of 1 µg/L (Figure 5C).

## 4. Discussion

Our study confirms previous results [38] showing that toxicity is not always linked to the level of exposure to pesticides. For I, H and IF, the highest mortalities were observed at the lowest concentration of 0.01 µg/L. For F and all the other mixtures, the highest mortalities were observed at intermediate concentrations of 0.1 and 1 µg/L. This is in line with previous data showing that chronic exposure to glyphosate and imidacloprid has a stronger impact on honey bee survival at low concentrations than at high concentrations [61,62,63]. Thus, relatively high exposure levels are not systematically those that induce the highest toxicity, and low exposure levels may induce toxicity comparable to or higher than that induced at high exposure levels. Such a bell-shaped dose-mortality profile observed in our study is called a nonmonotonic dose–effect relationship. These relationships are not uncommon, particularly with endocrine disruptors, and are also observed in the honey bee [64] and may involve different cellular and molecular mechanisms [65].

For a given pesticide mixture, the mode of interaction between pesticides strongly depends on the exposure level. Three-quarters of the pesticide mixture modalities induced subadditive, additive and synergistic effects (Table S1). IF induced an additive effect at 0.01, 0.1 and 10 µg/L and a synergistic effect at 1 µg/L. IH induced an additive effect at 0.01 and 0.1 µg/L and a synergistic effect at 1 and 10 µg/L. The ternary IHF mixture induced a subadditive effect at 0.01 and 10 µg/L, an additive effect at 1 µg/L and a synergistic effect at 0.1. The HF mixture was the only mixture that induced an antagonistic effect irrespective of the exposure concentration. Such a complex profile of interactions has been previously observed with mixtures associating EBI fungicides (prochloraz, propiconazole, fenbuconazole and myclobutanil) and the pyrethroid insecticide tau-fluvalinate [66], for which EBI fungicides elicited a synergistic effect with tau-fluvalinate at doses of 1 and 10 nmol/bee but an antagonistic effect at a dose of 0.1 nmol/bee. This antagonist action could be linked to the effect of EBI fungicides on cytochrome P450 (CYP450), which are considered the primary enzymes for the detoxification of phytochemicals [67] and pesticides [68] in honey bees. EBI fungicides, including difenoconazole, are not only potential inhibitors but also inducers of CYP450 [69,70,71]. Thus, the antagonistic effect of pesticide mixtures containing an EBI fungicide could be explained by the induction of detoxifying enzymes, which results in an increase in pesticide metabolism and a decrease in toxicity [66]. Such a mechanism of antagonism could be exemplified by piperonyl butoxide (PBO), an insecticide synergist that acts by inhibiting CYP450 [72] but that can also induce these enzymes [73], similar to EBI fungicides. This suggests that prolonged exposure or low-dose PBO and EBI fungicide could result in an induction of a number of genes coding for CYP450 [73,74]. The induction of CYP450 would consequently increase the metabolism of imidacloprid and glyphosate. However, the metabolism of imidacloprid by CYP450 generates metabolites that have similar or even higher toxicities than imidacloprid [75,76], whereas the metabolism of glyphosate generates less toxic metabolites such as amino-methylphosphonic acid (AMPA) [77]. Hence, the induction of CYP450 could explain both the increase in the toxicity of the IF mixtures and the antagonistic effects observed for the HF mixtures. Thus, the hypothesis of CYP450 induction by these pesticides deserves to be tested to increase the knowledge of interactions between substances in pesticide mixtures.

Exposure to pesticides, alone or in mixtures, did not modify the food consumption of honey bees. The absence of effects on food intake suggests that these pesticides do not exhibit particular repellent or attractive properties, at least at the evaluated concentrations, and that the different effects observed are not due to differences in exposure levels. For imidacloprid, this result is in accordance with the unchanged feeding behavior observed in summer bees exposed to imidacloprid for 10 days at concentrations ranging between 0.06 and 2 µg/L [78]. However, this result contrasts with the increased consumption of food containing neonicotinoids (including imidacloprid) by bees submitted for 24 h to a two-choice feeding assay [79]. These discrepancies in food consumption suggest that changes in food behavior are compensated for during long exposure periods. Lower food consumption has been observed with imidacloprid at a high concentration of 4.3 mg/L, but it could be due in part to the high exposure concentration and to the adjuvants of the product (Advise 2FL) used to prepare the feeding solution [80]. In contrast, no modification of food consumption was observed in bees exposed to glyphosate. This confirms the results of studies in which newly emerged bees were exposed for 14 days at a high concentration of 35 mg/L, and winter and summer honey bees were exposed for 22 days to glyphosate at 0.21 and 1.08 g/kg [77,80]. These results contrast with the higher preference of bees for food containing 10 µg/L glyphosate than for that containing 10 mg/L glyphosate [81]. However, it should be noted that a discrepancy between the present study and the previous work in which an increase in food consumption was observed in exposed bees [38]. The fact that an increase in food consumption is not systematically observed suggests that the physiological or toxicological status of bees could influence the effects of pesticides [82]. Thus, it cannot be ruled out that the bees used in the study of Almasri et al. 2020 were in energetic stress linked to wintering or to exposure to toxicants, which was magnified by the chemical stress induced by pesticides.

One of the possible causes of the pesticide effects in honey bees is the disturbance of the pro-oxidative/antioxidative balance. However, this cause has been scarcely explored for pesticide mixtures. Under normal physiological conditions, the antioxidant/pro-oxidant balance is in equilibrium. Pro-oxidants are mainly reactive oxygen species (ROS) that are permanently produced at moderate concentrations during mitochondrial respiration or as signaling mediators and defense molecules [83,84,85]. ROS can also be produced following exposure to toxicants, toxins, pollutants and radiation [86]. Oxidative damage occurs in the case of ROS overproduction or when there is a deficit in the antioxidant system, leading to possible alterations of lipids, proteins and DNA [87]. The antioxidant system is composed of nonenzymatic antioxidants, such as tocopherol and carotenoids, which could be diet-derived, and of antioxidant enzymes that can be modulated and act directly or indirectly on ROS [88,89]. Pesticides were previously reported to contribute to oxidative stress in plants and animals [90,91,92,93,94,95,96,97,98]. Imidacloprid and glyphosate were shown to induce oxidative stress in honey bees [78,99,100,101,102]. This led us to investigate whether imidacloprid, glyphosate and difenoconazole could induce oxidative stress and modulate antioxidative defenses and to determine whether these effects could be aggravated when honey bees were exposed to binary or ternary pesticide mixtures.

To assess the effect of the pesticides on oxidative stress, the activities of SOD, CAT, GPox, GR, GST and G6PDH were measured in surviving honey bees after 16 days of chronic oral exposure to pesticides. These enzymes work to limit oxidative stress, and they were previously shown to be modulated in honey bees under the pressure of chemical pesticides, spores of the biological pesticide *Bacillus thuringiensis* and environmental biotic stressors such as *Nosema* [53,90,103,104,105,106]. SOD, CAT and GPox are primary antioxidant enzymes that act directly on ROS. SOD transforms the highly reactive superoxide radical to the less reactive hydrogen peroxide and oxygen [107]. CAT converts hydrogen peroxide into water and oxygen [89]. GPox also acts on hydrogen peroxide and other organic hydroperoxides and catalyzes their reduction using electrons provided by GSH [108]. GR and G6PDH are secondary antioxidant enzymes. GR converts oxidized glutathione into its reduced form GSH [89]. G6PDH acts in the pentose phosphate pathway and generates NADPH, leading indirectly to the regeneration of reduced GSH [106]. GST, which could be considered a primary antioxidant enzyme, also plays a role in the detoxification process controlled by phase II enzymes. GST acts by conjugating GSH xenobiotics, which become more hydrophilic and therefore are transported outside of the organism [109,110]. GST also has a high affinity for lipid peroxidation products that are produced during oxidative stress, and transform them into less toxic hydroxyl derivatives [89,109].

Honey bees exposed to pesticides at 1 µg/L exhibited large variations in antioxidant enzyme activities. As shown by PCA, the activities of GST and GPox in the midgut were positively correlated with each other, as well as the activities of GPox, GST and GR in the head. The activities of head and midgut GST and head GPox varied greatly and represented at least 566%, 223% and 225% of the control activities in all exposure groups, respectively, except for the HF exposure group, which did not exhibit different activities for these enzymes from those in the control group. A similar increase in GST activity was previously observed when honey bees were exposed to imidacloprid and other neonicotinoids, such as thiamethoxam [53,104]. The increase in GST and GPox activities strongly reflected the attempts of the organism to counteract the oxidative stress that took place following the exposure to pesticides. In addition, an increase in GST activity may reflect the activation of the detoxification process through the conjugation of xenobiotics with glutathione [110]. The activities of GST and GPox rely on the presence of reduced glutathione, which is under the control of GR, and GR uses NADPH as a reducer (produced in large part by G6PDH). However, an increase in the activity of GR in the head (at least 300% of that in the control) and G6PDH in the midgut (at least 782% of the control activity) was observed in almost all exposure conditions. In the midgut, the concomitant increases in GST and G6PDH activities correlated well because G6PDH generates the NADPH necessary for the reduction of oxidized glutathione into its reduced form for use by GST. Consequently, the activity of enzymes responsible for the destruction of peroxides (GST and GPox) increased in correlation with the increasing activities of enzymes (GR and G6PDH) responsible for the regeneration of cofactors (GSH and NADPH) necessary for the functioning of GST and GPox.

In contrast to that, at the dose of 1 µg/L, the change in antioxidant enzyme activities was more complex and less pronounced at 0.1 µg/L. This suggested either that the oxidative stress was less pronounced at 0.1 µg/L or that the honey bees were able to recover from some of the stress. To distinguish between these two hypotheses, additional markers of oxidative stress were investigated in the exposure groups at 0.1 µg/L through the measurement of lipid peroxidation and protein carbonylation. In general, lipid peroxidation (except for HF) and protein carbonylation (except for IF, IH, HF and IHF) decreased in all exposure groups to below normal physiological rates. This indicated that the antioxidant systems were likely highly induced at the 0.1 µg/L exposure level to be able to reduce lipid and protein oxidations below the normal physiological rates.

Exposure to H and F alone induced pronounced variations in the antioxidant enzymes at 1 µg/L, and the levels of lipid peroxidation and protein carbonylation were below the physiological levels observed at 0.1 µg/L. However, the HF mixture induced the lowest variations in antioxidant enzyme activities at 0.1 and 1 µg/L, and the levels of lipid peroxidation and protein carbonylation at 0.1 µg/L were similar to normal physiological levels. Therefore, the oxidative stress triggered by H and F was abolished when both pesticides were mixed together. In the mixture, the loss of the oxidative stress induced by H might be explained by an induction of CYP450 by difenoconazole (F) [69,70,71], leading to the detoxification of glyphosate and the reduction in oxidative stress and toxicity of the mixture. However, this hypothesis does not explain why, in the mixture, the oxidative stress induced by F was also inhibited. This exemplifies that the mechanism of action of a given mixture does not merely correspond to the sum of the modes of action of each substance, as was previously shown for the interaction between the fungicide difenoconazole, the herbicide glyphosate and the insecticide imidacloprid [82].

Changes in GR and GPox activities were observed in the head, while changes in CAT, SOD and GST activities were observed in both the head and midgut. Therefore, the effects of the pesticides were not restricted to the midgut, which is the primary site of oral exposure, but they were also extended to all biological compartments, leading to systemic oxidative stress that could compromise bee health. This systemic action not only reflected the distribution of the substances in the whole body, as already observed with imidacloprid [111] but also showed that all tissues are sensitive to oxidative stress. In addition, for the same enzyme and the same type of exposure, physiological responses to pesticides may be tissue specific. This was the case for SOD at an exposure level of 1 µg/L, whose activity decreased in the head and increased in the midgut, and for CAT and GST at exposure levels of 1 µg/L and 0.1 µg/L, respectively, whose activities were not modulated in the head but decreased in the midgut.

Numerous studies have shown that imidacloprid (e.g., [112,113,114,115,116]) and glyphosate [117,118,119,120,121,122] induce adverse sublethal effects in honey bees at the behavioral and physiological levels. It is noteworthy that imidacloprid elicits sublethal effects at exposure levels for which no interaction with its primary target (the nicotinic acetylcholine receptors [123]), responsible for the lethal insecticide effect, could be expected. However, although the toxicity of imidacloprid to an insect, such as the honey bee, could be in part predictable on the basis of its insecticidal activity, it appears obvious that glyphosate does not induce its toxicity to the honey bee by its interaction with its plant target EPSPS because this enzyme does not exist in animals [42]. Concerning the triazole fungicide difenoconazole, its molecular target, fungal lanosterol 14α-demethylase, is a cytochrome P-450-dependent enzyme that can be inhibited by azole fungicides, as all cytochrome P-450s in animals [124]. Hence, the effects of difenoconazole linked to its inhibiting action on cytochrome P-450s may easily be expected in animals, in addition to its other sublethal effects observed in bees not closely related to its action on cytochrome P-450s [38,51,82,125,126,127]. Thus, the occurrence of sublethal effects reveals that imidacloprid, glyphosate and difenoconazole possess different biological targets responsible for their numerous effects. Consequently, the different targets of imidacloprid, glyphosate and difenoconazole offer a pathway interaction network that could account for the antagonistic, additive and synergistic effects of the mixtures of pesticides that can occur at different exposure levels and with different mixture combinations. This interaction network could be widely extended by considering that imidacloprid is metabolized in the honey bee into at least six metabolites that are toxic at low exposure levels [75,76] and that glyphosate is metabolized into at least five metabolites in animals [128], including the environmental AMPA metabolite [129].

## 5. Conclusions

The present study confirms previous results showing that chronic oral exposure to environmental concentrations of insecticides, fungicides and herbicides could negatively affect the survival of winter honey bees [38]. The toxicity of the pesticides highly increased when they occurred as mixtures, and the highest mortalities were recorded at intermediate exposure concentrations of 0.1 and 1 µg/L. Our data showed that the oxidative balance was severely disrupted by pesticides, both alone and in mixtures. The induction of oxidative stress could be one of the prevalent mechanisms that could explain the toxicity of pesticide mixtures. Hence, it is reasonable to propose that the adverse effects of exposure to pesticides on survival and oxidative stress could be aggravated by the cold and humid conditions of the winter season. Additionally, the presence of residues of numerous pesticides in beehive matrices [130] could explain, at least in part, the increase in winter colony losses observed in many countries [131].

## Data Availability

The datasets used and/or analyzed during the current study are available from the corresponding author on reasonable request.

## Supplementary Materials

The following supporting information can be downloaded at: https://www.mdpi.com/article/10.3390/toxics10030104/s1, Table S1. Modes of interaction of the different pesticide combinations and their effects on honey bee mortality; Table S2. Overall comparison of the effects of pesticides on mortalities; Table S3. Effects of exposure to pesticides on honey bee food consumption; Table S4. Effects of exposure to pesticides, alone or in combination, at 0.1 µg/L on physiological markers in winter honey bees; Table S5. Effects of exposure to pesticides, alone or in combination, at 1 µg/L on physiological markers in winter honey bees; Table S6. Effects of exposure to pesticides, alone or in combination, at 0.1 µg/L on lipid peroxidation and protein carbonylation.
