# Supplementary material for: Toxicity of the Pesticides Imidacloprid, Difenoconazole and Glyphosate Alone and in Binary and Ternary Mixtures to Winter Honey Bees: Effects on Survival and Antioxidative Defenses
Source: Toxics. 2022 Feb 23;10(3):104. doi: 10.3390/toxics10030104 (PMC8954695; doi:10.3390/toxics10030104)
Supplement: Supplementary file 1 [file toxics-10-00104-s001.zip › toxics-1601930-supplementary.pdf]

toxics MDPI

Supplementary Materials: Toxicity of the Pesticides Imidacloprid, Difenoconazole and Glyphosate Alone and in Binary and Ternary Mixtures to Winter Honey Bees: Effects on Survival and Antioxidative Defenses

Elisa Pal, Hanine Almasri, Laurianne Paris, Marie Diogon, Maryline Pioz, Marianne Cousin, Déborah Sené, Sylvie Tchamitchian, Daiana Antonia Tavares, Frédéric Delbac, Nicolas Blot, Jean-Luc Brunet and Luc P. Belzunces

Table S1. Modes of interaction of the different pesticide combinations and their effects on honey bee mortality.

Treatment		Mortality rate (%) ± SD	Corrected Mortality	Expected Mortality	IR	Statistical significance ($p < 0.05$)	Mode of Interaction
0 µg/L	C	8.57 ± 2.52	0.00	-	-	a	-
0.01 µg/L	I	27.62 ± 5.68	19.05	-	-	b	-
	H	18.38 ± 2.52	9.81	-	-	ac	-
	F	26.67 ± 3.56	18.10	-	-	b	-
	IF	38.33 ± 3.28	29.76	37.15	0.80	d	Additivity
	IH	36.00 ± 2.98	27.43	28.86	0.95	d	Additivity
	HF	15.24 ± 3.71	6.67	27.91	0.24	c	Antagonism
	IHF	37.62 ± 5.44	29.05	46.96	0.62	d	Sub- additivity
0.1 µg/L	I	26.19 ± 2.52	17.62	-	-	b	
	H	23.33 ± 1.26	14.76	-	-	b	
	F	20.48 ± 3.71	11.91	-	-	b	
	IF	37.62 ± 2.62	29.05	29.53	0.98	c	Additivity
	IH	35.24 ± 3.85	26.67	32.38	0.82	c	Additivity
	HF	22.86 ± 1.63	14.29	26.67	0.54	b	Antagonism
	IHF	66.19 ± 3.17	57.62	44.29	1.30	d	Synergism
1 µg/L	I	13.81 ± 1.78	5.24	-	-	b	-
	H	27.65 ± 2.52	19.08	-	-	b	-
	F	21.90 ± 3.17	13.33	-	-	b	-
	IF	35.24 ± 1.78	26.67	18.57	1.44	c	Synergism
	IH	54.76 ± 3.25	46.19	24.32	1.90	d	Synergism
	HF	19.05 ± 1.78	10.48	32.41	0.32	b	Antagonism
	IHF	49.05 ± 2.72	40.48	37.65	1.08	d	Additivity
10 µg/L	I	15.71 ± 2.62	7.14	-	-	b	-
	H	25.24 ± 3.71	16.67	-	-	c	-
	F	20.48 ± 2.30	11.91	-	-	bc	-
	IF	29.44 ± 1.72	20.87	19.05	1.10	d	Additivity
	IH	40.67 ± 2.98	32.10	23.81	1.35	d	Synergism
	HF	21.43 ± 2.30	12.86	28.58	0.45	bc	Antagonism
	IHF	30.48 ± 6.59	21.91	35.72	0.61	d	Sub- additivity

Winter honey bees were fed sucrose solutions containing no pesticides (C), difenoconazole (F), glyphosate (H), glyphosate + difenoconazole (HF), imidacloprid (I), imidacloprid + difenoconazole (IF), imidacloprid + glyphosate + difenoconazole (IHF) or imidacloprid + glyphosate (IH) at 0.01, 0.1, 1 and 10 µg/L for 16 days. The data represent the mean mortality rate (%) of 14 repetitions ± standard deviation (SD), which is the mortality corrected by the control and the expected mortality (which is the sum of the corrected mortality of each pesticide alone). The interaction ratio (IR) was calculated by dividing the corrected mortality by the expected mortality. Data with different letters are significantly different ($p < 0.05$).

Table S2. Overall comparison of the effects of pesticides on mortalities.

Treatment	Corrected Mortality (%)	Statistical significance (p < 0.05)
C	0.00	a
I1	5.24	b
HF0.01	6.67	b
I10	7.14	b
H0.01	9.81	b
HF1	10.48	b
F0.1	11.91	bc
F10	11.91	bc
HF10	12.86	bc
F1	13.33	bc
HF0.1	14.29	bc
H0.1	14.76	bc
H10	16.67	bc
I0.1	17.62	bc
F0.01	18.10	bc
I0.01	19.05	bc
H1	19.08	bc
IF10	20.87	c
IHF10	21.91	cd
IH0.1	26.67	cd
IF1	26.67	cd
IH0.01	27.43	cd
IHF0.01	29.05	cd
IF0.1	29.05	cd
IF0.01	29.76	cd
IH10	32.10	cd
IHF1	40.48	d
IH1	46.19	de
IHF0.1	57.62	e

Winter honey bees were fed sucrose solutions containing no pesticides (C, control), imidacloprid (I), difenoconazole (F), glyphosate (H), glyphosate + difenoconazole (HF), imidacloprid + difenoconazole (IF), imidacloprid + glyphosate (IH) or imidacloprid + glyphosate + difenoconazole (IHF) at 0.01, 0.1, 1 and 10 µg/L for 16 days. The data represent the mean corrected mortality rate (%) of 14 repetitions ± standard deviation (SD). The significant differences between mortalities induced by the different treatments at the four different concentrations were determined. Numbers after the abbreviations of each treatment refer to the concentrations of the pesticides in the sucrose solution. Data with different letters are significantly different ($p < 0.05$).

Table S3. Effects of exposure to pesticides on honey bee food consumption.

	Treatment	Cumulated food consumption ± SD (mg/bee)	Daily food consumption (mg/bee)	Daily substance consumption (ng/bee)	Statistical significance ($p < 0.05$)
0 µg/L	C	852.31 ± 48.19	53.27	0	abc
0.01 µg/L	F	822.97 ± 89.15	51.44	$4.2\ 10^{-04}$	abcd
	H	753.71 ± 40.43	47.11	$3.8\ 10^{-04}$	d* (*p*=0.0496)
	I	840.41 ± 51.13	52.53	$4.3\ 10^{-04}$	abcd
	HF	854.67 ± 38.07	53.42	$4.3\ 10^{-04}$	abc
	IF	864.53 ± 62.74	54.03	$4.4\ 10^{-04}$	abc
	IH	792.96 ± 70.45	49.56	$4.0\ 10^{-04}$	abcd
	IHF	799.77 ± 34.13	49.99	$4.0\ 10^{-04}$	bcd
	F	789.16 ± 58.95	49.32	$4.0\ 10^{-03}$	bcd
	H	819.34 ± 49.23	51.21	$4.1\ 10^{-03}$	abcd

0.1 µg/L	I	859.14 ± 64.94	53.70	4.3 10^{-03}	abc
	HF	788.80 ± 153.85	49.23	4.0 10^{-03}	abcd
	IF	812.24 ± 44.42	50.77	4.1 10^{-03}	abcd
	IH	818.51 ± 49.92	51.16	4.1 10^{-03}	abcd
	IHF	809.04 ± 120.65	50.57	4.1 10^{-03}	bcd
1 µg/L	F	783.40 ± 29.47	48.96	4.0 10^{-02}	cd
	H	798.14 ± 82.04	49.88	4.0 10^{-02}	bcd
	I	834.75 ± 52.66	52.17	4.2 10^{-02}	abcd
	HF	836.89 ± 68.49	52.31	4.2 10^{-02}	abcd
	IF	795.09 ± 113.68	49.69	4.0 10^{-02}	abcd
	IH	851.14 ± 85.00	53.20	4.3 10^{-02}	abcd
	IHF	813.25 ± 24.33	50.83	4.1 10^{-02}	abcd
10 µg/L	F	778.52 ± 47.31	48.66	3.9 10^{-01}	bcd
	H	917.31 ± 48.92	57.33	4.6 10^{-01}	a
	I	831.87 ± 46.26	51.99	4.2 10^{-01}	abcd
	HF	871.48 ± 23.42	54.47	4.4 10^{-01}	ab
	IF	790.29 ± 122.04	49.39	4.0 10^{-01}	bcd
	IH	851.09 ± 86.01	53.19	4.3 10^{-01}	abcd
	IHF	774.66 ± 83.93	48.42	3.9 10^{-01}	bcd

Winter honey bees were fed sucrose solutions containing no pesticides (C), difenoconazole (F), glyphosate (H), imidacloprid (I), glyphosate + difenoconazole (HF), imidacloprid + difenoconazole (IF), imidacloprid + glyphosate (IH), or imidacloprid + glyphosate + difenoconazole (IHF) at 0.01, 0.1, 1 and 10 µg/L for 16 days. Food consumption was evaluated during the 16 days by measuring the food consumed daily by the bees alive in each of the 14 cages per treatment. The cumulative food consumption is expressed in milligrams per bee (mg/bee) ± standard deviation (SD). Statistical analyses were performed using the Kruskal-Wallis test followed by pairwise comparisons using the Wilcoxon rank sum test with Benjamini-Hochberg correction. Asterisks indicate significant differences from the control group (* $p \leq 0.05$).

Table S4. Effects of exposure to pesticides, alone or in combination, at 0.1 µg/L on physiological markers in winter honey bees.

Physiological marker (mAU/min/g of tissue)	Control	I [0.1 µg/L]	F [0.1 µg/L]	H [0.1 µg/L]	IF [0.1 µg/L]	IH [0.1 µg/L]	HF [0.1 µg/L]	IHF [0.1 µg/L]
**SOD$_{(h)}$**	7.288 ± 1.090 ^{b}	6.576 ± 3.067 ^{b}	12.184 ± 4.446 ^{a} ↑	7.733 ± 3.960 ^{b}	7.733 ± 5.636 ^{b}	8.119 ± 3.960 ^{ab}	7.809 ± 0.843 ^{ab}	7.733 ± 3.960 ^{b}
**CAT$_{(h)}$**	3.328 ± 0.331 ^{b}	1.012 ± 0.064 ^{d} ↓	1.769 ± 1.037 ^{d} ↓	2.940 ± 0.229 ^{cd} ↓	3.233 ± 0.350 ^{bc}	3.143 ± 0.223 ^{bc}	3.913 ± 0.337 ^{a} ↑	3.086 ± 0.331 ^{bc}
**GST$_{(h)}$**	13.000 ± 1.560	12.090 ± 1.430	12.350 ± 3.250	13.650 ± 2.080	14.430 ± 1.170	13.910 ± 0.910	14.690 ± 0.910	14.170 ± 0.910
**GR$_{(h)}$**	2.721 ± 0.700 ^{ac}	3.057 ± 1.049 ^{ac}	1.389 ± 0.048 ^{a}	3.195 ± 0.481 ^{ac}	1.701 ± 0.982 ^{ab}	2.268 ± 0.491 ^{ac}	3.067 ± 0.432 ^{bc}	3.969 ± 0.982 ^{c}
**GPox$_{(h)}$**	3.297 ± 0.938 ^{ab}	7.060 ± 0.733 ^{c} ↑	2.804 ± 0.794 ^{a}	3.675 ± 0.648 ^{ab}	3.481 ± 0.794 ^{ab}	2.998 ± 1.048 ^{a}	3.971 ± 0.464 ^{ab}	4.714 ± 0725 ^{b}
**SOD$_{(m)}$**	6.972 ± 0.768 ^{ab}	8.841 1.837 ^{bc}	4.585 ± 1.774 ^{a}	8.022 ± 2.807 ^{ab}	11.789 ± 1.837 ^{cd} ↑	15.472 ± 3.606 ^{de} ↑	5.026 ± 1.218 ^{a}	17.765 ± 2.625 ^{e} ↑
**G6PDH$_{(m)}$**	2.040 ± 0.000 ^{ab}	1.837 ± 0.495 ^{b}	3.422 ± 0.624 ^{a}	3.193 ± 0.578 ^{ab}	2.681 ± 0.465 ^{ab}	2.138 ± 0.640 ^{ab}	2.312 ± 0.765 ^{ab}	1.807 ± 0.319 ^{b}
**CAT$_{(m)}$**	0.997 ± 0.051 ^{ab}	0.441 ± 0.092 ^{c} ↓	1.443 ± 0.263 ^{a}	1.050 ± 0.188 ^{ab}	0.863 ± 0.139 ^{ab}	0.776 ± 0.158 ^{bc}	0.836 ± 0.283 ^{b}	0.803 ± 0.137 ^{bc}
**GST$_{(m)}$**	0.347 ± 0.082 ^{a}	0.039 ± 0.012 ^{b} ↓	0.053 ± 0.014 ^{b} ↓	0.044 ± 0.008 ^{b} ↓	0.432 ± 0.250 ^{a}	0.259 ± 0.087 ^{a}	0.286 ± 0.066 ^{a}	0.385 ± 0.181 ^{a}
**G6PDH$_{(a)}$**	22.789 ± 7.608	28.797 ± 11.885	19.267 ± 0.878	17.995 ± 3.349	29.004 ± 6.255	8.151 ± 8.151	19.000 ± 5.216	24.032 ± 7.031

Winter honey bees were fed sucrose solutions containing no pesticides (C), difenoconazole (F), glyphosate (H), imidacloprid (I), glyphosate + difenoconazole (HF), imidacloprid + difenoconazole (IF), imidacloprid + glyphosate (IH), or imidacloprid + glyphosate + difenoconazole (IHF) at 0.01, 0.1, 1 and 10 µg/L for 16 days. A multiple marker approach was

performed to study the effects of these pesticides at 0.1 µg/L on oxidative stress. SOD, CAT and GST activities were measured in the head (h) and midgut (m). GPox and GR were measured in the head (h), whereas G6PDH was measured in the midgut (m) and abdomen (a). Seven samples (n=7) of 3 tissues were collected in each treatment, and the mean value of enzymatic activity was calculated for each treatment. The enzymatic activity was expressed in milliunits of absorbance per minute and per g of tissue (mAU/min/g of tissue) ± standard deviation (SD). Data with different letters are significantly different ($p < 0.05$). Arrows indicate an increase (↑) or a decrease (↓) in activity relative to that in the control group.

Table S5. Effects of exposure to pesticides, alone or in combination, at 1 µg/L on physiological markers in winter honey bees.

Physiological marker (mAU/min/g of tissue)	Control	I [1 µg/L]	F [1 µg/L]	H [1 µg/L]	IF [1 µg/L]	IH [1 µg/L]	HF [1 µg/L]	IHF [1 µg/L]
**SOD(h)**	7.288 ± 1.090 ^{a}	4.081 ± 0.000 ^{d} ↓	4.897 ± 1.117 ^{cd} ↓	6.122 ± 0.000 ^{b} ↓	6.122 ± 0.000 ^{bc} ↓	3.061 ± 1.443 ^{d} ↓	7.529 ± 0.678 ^{a}	3.410 ± 0.000 ^{d} ↓
**CAT(h)**	3.328 ± 0.331 ^{ab}	3.639 ± 1.224 ^{b}	2.143 ± 0.573 ^{a}	2.333 ± 0.284 ^{a}	2.154 ± 0.219 ^{a}	2.636 ± 0.313 ^{ab}	3.070 ± 0.488 ^{ab}	2.517 ± 0.000 ^{ab}
**GST(h)**	13.000 ± 1.560	92.517 ± 6.908 ^{de} ↑	73.605 ±6.497 ^{b} ↑	81.519 ± 4.741 ^{bc} ↑	90.930 ± 4.100 ^{cd} ↑	104.082 ± 0.962 ^{e} ↑	14.318 ± 0.550 ^{a}	100.680 ± 0.000 ^{de} ↑
**GR(h)**	2.721 ± 0.700 ^{a}	9.455 ± 1.369 ^{b} ↑	9.659 ± 1.825 ^{b} ↑	8.163 ± 1.053 ^{b} ↑	10.544 ± 0.589 ^{b} ↑	10.884 ± 0.000 ^{b} ↑	2.572 ± 0.969 ^{a}	9.523 ± 0.000 ^{b} ↑
**GPox(h)**	3.297 ± 0.938 ^{a}	7.755 ± 0.912 ^{bc} ↑	9.244 ± 1.707 ^{bd} ↑	7.426 ± 0.871 ^{b} ↑	9.870 ± 1.558 ^{cd} ↑	10.877 ± 0.000 ^{d} ↑	3.595 ± 0.444 ^{a}	9.530 ± 0.000 ^{bd} ↑
**SOD(m)**	6.972 ± 0.768 ^{a}	16.727 ± 3.866 ^{bc} ↑	14.142 ± 1.513 ^{b} ↑	19.551 ± 3.078 ^{c} ↑	19.040 ± 2.699 ^{bc} ↑	15.816 ± 0.721 ^{bc} ↑	4.384 ± 0.848 ^{a}	18.367 ± 0.000 ^{bc} ↑
**G6PDH(m)**	2.040 ± 0.000 ^{a}	11.564 ± 2.356 ^{ab}	21.315 ± 3.086 ^{bc} ↑	15.963 ± 4.114 ^{bc} ↑	19.954 ± 7.080 ^{bc} ↑	23.469 ± 4.329 ^{c} ↑	2.892 ± 0.659 ^{a}	25.850 ± 0.000 ^{c} ↑
**CAT(m)**	0.997 ± 0.051 ^{c}	0.570 ± 0.117 ^{ab} ↓	0.481 ± 0.104 ^{ab} ↓	0.486 ± 0.079 ~~a~~ ↓	0.545 ± 0.043 ^{ab} ↓	0.833 ± 0.014 ^{c}	0.618 ± 0.042 ^{ab} ↓	0.762 ± 0.000 ^{bc}
**GST(m)**	0.347 ± 0.082 ^{a}	0.776 ± 0.061 ^{b} ↑	1.306 ± 0.143 ^{c} ↑	1.102 ± 0.163 ^{c} ↑	1.245 ± 0.163 ^{c} ↑	1.674 ± 0.061 ^{d} ↑	0.286 ± 0.061 ^{a}	2.204 ± 0.000 ^{e} ↑
**G6PDH(a)**	22.789 ± 7.608	21.428 ± 3.818	18.877 ± 3.893	17.346 ± 4.929	21.768 ± 4.329	30.782 ± 26.696	18.448 ± 4.873	43.537 ± 0.000

Winter honey bees were fed sucrose solutions containing no pesticides (C), difenoconazole (F), glyphosate (H), imidacloprid (I), glyphosate + difenoconazole (HF), imidacloprid + difenoconazole (IF), imidacloprid + glyphosate (IH), or imidacloprid + glyphosate + difenoconazole (IHF) at 0.01, 0.1, 1 and 10 µg/L for 16 days. A multiple marker approach was performed to study the effects of these pesticides at 1 µg/L on oxidative stress. SOD, CAT and GST were measured in the head (h) and midgut (m). GPox and GR were measured in the head (h), whereas G6PDH was measured in the midgut (m) and abdomen (a). Seven samples (n=7) of 3 tissues were collected in each treatment, and the mean value of enzymatic activity was calculated for each treatment. The enzymatic activity was expressed in milliunits of absorbance per minute and per g of tissue (mAU/min/g of tissue) ± standard deviation (SD). Treatments with different letters are significantly different ($p < 0.05$). Arrows indicate an increase (↑) or a decrease (↓) in activity relative to that in the control group.

Table S6. Effects of exposure to pesticides, alone or in combination, at 0.1 µg/L on lipid peroxidation and protein carbonylation.

Physiological marker	Unit	Control	I [0.1 µg/L]	F [0.1 µg/L]	H [0.1 µg/L]	IF [0.1 µg/L]	IH [0.1 µg/L]	HF [0.1 µg/L]	IHF [0.1 µg/L]
**TBARS**	µM MDA/mg of tissue	0.564 ± 0.126 ^{a}	0.358 ± 0.061 ^{b} ↓	0.302 ± 0.128 ^{bc} ↓	0.217 ± 0.053 ^{C} ↓	0.144 ± 0.040 ^{d} ↓	0.167 ± 0.057 ^{d} ↓	0.657 ± 0.112 ^{a}	0.122 ± 0.030 ^{d} ↓
**Protein carbonylation**	% of carbonylated proteins/mg of tissue	17.886 ± 4.847 ^{cd}	7.411 ± 2.754 ^{a} ↓	10.535 ± 2.822 ^{ab} ↓	9.418 ± 5.750 ^{ab} ↓	28.243 ± 4.088 ^{e} ↑	13.302 ± 3.769 ^{bc}	24.633 ± 4.086 ^{de}	17.028 ± 3.206 ^{c}

Winter honey bees were fed sucrose solutions containing no pesticides (C), difenoconazole (F), glyphosate (H), imidacloprid (I), glyphosate + difenoconazole (HF), imidacloprid + difenoconazole (IF), imidacloprid + glyphosate (IH), or imidacloprid + glyphosate + difenoconazole (IHF) at 0.01, 0.1, 1 and 10 µg/L for 16 days. Lipid peroxidation and protein carbonylation were measured in the midguts of honey bees exposed to those pesticides at 0.1 µg/L. Nine samples of 3 midguts (n=9) were collected in each treatment for the measurement of lipid peroxidation, whereas 12 samples of 1 midgut (n=12) were collected for protein carbonylation. Lipid peroxidation and protein carbonylation were expressed in µmoles

of malondialdehyde per mg of tissue (µmol of MDA/mg of tissue) and percentage of carbonylated proteins per mg of tissue (% of carbonylated proteins/mg of tissue) ± standard deviation (SD). Treatments with the different letters are significantly different ($p < 0.05$). Arrows indicate an increase (↑) or a decrease (↓) in activity relative to that in the control group.
